# The Distal Phenomena That Accompany High Arterial Tension
*A Paper read to the Bath and Bristol Branch of the British Medical Association on 31st October, 1928.


**Published:** 1929

**Authors:** Carey F. Coombs

**Affiliations:** Physician, Bristol General Hospital


### THE DISTAL PHENOMENA THAT ACCOMPANY HIGH ARTERIAL TENSION.*

BY

CAREY F. COOMBS, M.D., F.R.C.P.,

*Physician, Bristol General Hospital.*

---

No one who studies the diseases of the cardio-vascular system can fail to note the diversity of the cerebral symptoms that may accompany high arterial tension.

Looking back through case-notes of patients with high blood pressure, I was struck afresh by this diversity and at the same time by the parallelism between these cerebral phenomena and those seen in other organs in the presence of high tension. These parallelisms are set forth in the following table :—

*Distal Phenomena Associated with High Blood Pressure.*

| ACUTE, WITH RAPID RECOVERY. | ACUTE, WITH PARTIAL OR NO RECOVERY. | PROGRESSIVE. |
|---|---|---|
| | *Brain.* | |
| Recurrent aphasia, hemiplegia, etc. | Thrombosis, softening (hemorrhage). | Dementia, paresis (of type described). |
| | *Heart.* | |
| " Ischæmia cordis," or angina of effort. | Coronary thrombosis, or cardiac infarction. | Myofibrosis cordis or cardiosclerosis. |
| | *Limb.* | |
| Intermittent claudication. | Gangrene. | Gangrene. |
| | *Bowel.* | |
| (Case recorded.) | Mesenteric thrombosis. | ? |

* A Paper read to the Bath and Bristol Branch of the British Medical Association on 31st October, 1928.

These distal effects of high pressure are of great interest, for example, in relation to the muscular functions of the wall of the heart and of the lower limb. The analogies between these two have been stressed for over a century, even to the point of labelling the one set of phenomena "angina cruris," so as to bring them into line with those of "angina pectoris." But it is only in recent years that recognition of the symptoms of cardiac infarction has rehabilitated the coronary hypothesis of angina, and in so doing has thrown a new light on the meaning of the anginal attack, whether in the leg or in the heart. It has thus become clear that persons with high arterial tension are subject to two kinds of interference with muscular function, whether in the wall of the heart or in the calf of the leg: a transient kind of attack, in which pain is the prominent symptom; and a more lasting affair, which involves permanent injury of the parts affected. So far as the heart is concerned we are able, and indeed constrained, to add a third category, that of the slowly progressive degradation of muscle function, that is all the more striking because it is imposed on a cardiac wall from which the high pressure is exacting a supernormal effort.

Now, if we examine the objective cerebral phenomena that accompany high blood pressure, we shall also find the same three groups. The interference with function may be abrupt or it may be gradual; and if it is abrupt it may pass off leaving the patient none the worse, or it may inflict on him a permanent injury.

Examples of this kind are familiar enough, and the text-book descriptions are explicit. We need not stop to discuss these except to remark that it is doubtful whether it is so easy to make a diagnosis between hemorrhage and thrombosis as we have been led to believe. It seems to me that it is almost as unsafe to exclude a diagnosis of hemorrhage because the blood pressure has been low as it is to exclude a diagnosis of thrombosis because the pressure has been high. Probably few would cavil at the suggestion that a stroke in which the patient is not deprived of consciousness is more likely to be due to blocking than to bursting of an artery; and there is a turn of opinion towards accepting a similar explanation of most strokes. Dr. James Collier[1] says we should regard as thrombotic most strokes that are survived for ten days after the onset. There is, of course, an irreducible group of strokes of which hemorrhage is the only explanation, and moreover there are plenty of sites—the retina, the mucous membranes, and so on—where hemorrhages do occur in persons with high blood pressure. Yet there is also in the hyperpietic a thrombotic type of stroke, leading to necrosis of the parts deprived of their blood, just as a similar process may in the leg end in gangrene and in the heart wall in infarction.

Such phenomena as these are not difficult to understand. But what are we to say of the attacks that come and go, leaving no permanent organic results as far as can be seen?

Take, for example, the case of a friend of mine, with high arterial tension; a man accustomed to work to the limit of his strength and beyond it, till at length he was seriously disturbed to find himself unable to walk briskly, even on the level, without provoking oppressive pain under the sternum.

This went on for months, till he consented to a restriction of his undertakings, some years ago, and I do not believe he ever gets the pain now ; nor has he developed any other evidence of cardiac disease. Yet I have no doubt that the pain was cardiac.

The cerebral counterpart of this, recurrent paresis, has been described by many physicians. A brief note of the first case I saw, twenty-five years ago, may suffice as an illustration.

A man of sixty was admitted to St. Mary's Hospital unconscious, and in a right-sided convulsion. Seen two days later, he showed weakness in his right arm, a little in his right leg, a tendency to recurrent utterance, and some lingering confusion of mind. In an attack two years earlier he had been admitted in much the same state, and a few weeks later he came in again at the end of an attack.

More often there is a hemiplegia, for all the world like the usual capsular lesion, except that it is transient. Sometimes it is an aphasic and sometimes a hemianopic syndrome that recurs. Sometimes the series is closed by an attack that inflicts permanent damage, but in many instances the attacks return over a period of years and no final breakdown occurs. In 1909 Dr. George Parker, writing in our *Journal*,[2] described these cases, and felt himself unable to support the view still held by many physicians that an arterial spasm is responsible. This conclusion of Dr. Parker's Clifford Allbutt quoted with approval in his book on *Diseases of the Arteries.*[3]

It would be easy to allow this fascinating branch of our subject to divert us from the main thesis, but if we are to get a general view of these distal phenomena we must also scrutinize other parts of the circulation. There is no better example of these transient interferences with function than intermittent limp.

An instance of this is furnished by a hospital patient, a man aged 60 with a high arterial tension, who was already subject to the angina of effort, when he began to notice pain in the right calf coming on after he had walked 300 yards and rendering further progress impossible. The systolic blood pressure in one arm is 240 and in the other 185. In the legs it is 180 to 190. Drs. Beatson and Hooker found that exertion made no difference to the blood pressure in either arm, while amyl nitrite brought both down by one-third.

There are, then, in persons with high arterial tension, interferences with function, alike in their course, whether brain, heart or limb be involved; abrupt in onset, brief in duration, leaving no trace of permanent injury.

Next we have briefly to discuss certain steadily progressive degradations of function that are also found in hyperpietics. The issue is confused in the cardiac branch of this subject; for who is to judge how much of the myocardial failure, that so often closes the career of the hyperpietic, should be ascribed to the excessive demand laid on the cardiac muscle by the high pressure, and how much to failure of the coronary function? How much of the failure is proximal, and how much distal? In the limb I do not think we often see progressive decrease of ability in the type of arterial disease that we are discussing, short of senile gangrene, which develops slowly in some instances. It is in the brain that we can most clearly discern the gradual type of failure. The kind of thing I am thinking of may best be illustrated by a brief note of one of the half-dozen examples that have come under my observation. For these the term "hypertensive encephalopathy," used by Oppenheimer and Fishberg,[4] seems appropriate.

A man aged 59 had a fit four years ago, and was found to have retinal changes ; yet a year later he was well enough to get a good prognosis from a careful physician. Soon after this a renal epistaxis occurred. Recently he has developed an unsteady gait, emotionalism, a tendency to repeat himself in speech, and uncertain control of his anal sphincter. His arteries are thick and hard, his blood pressure 220/140, his aortic second sound loud. An aortic diastolic murmur is heard at the lower end of the sternum. He has a shuffling, unsteady gait, without true ataxia ; his deep reflexes are increased, and the plantar response on the left side is extensor. The retinal vessels are blurred in one or two spots by patches of exudate. The pupils, though small, react naturally. He is childish and emotional, but not boastful ; and although his speech is slurred, there is no tremor of lips or tongue. The urine is of a low specific gravity and contains albumin. There is good reason to believe that he was never infected with syphilis.

This is a fair sample of the progressive degeneration of the brain that may be seen in chronic hyperpiesis. It is true that it fades off into the syndrome of sudden catastrophe.

A good example of this is seen in the case of a man aged 50 who two years earlier had had a stroke without loss of consciousness, which deprived him of power in his left side. From this he recovered, though it left him clumsy on that side. Some months after this he got another attack implicating his speech and paralysing his right side, from which he made no recovery. Mental deterioration set in, though he seemed worse in this respect than he was actually, because his emotional expression was disproportionately affected, and he laughed and cried on the least provocation. He showed a spastic state and could scarcely stand. His cerebro-spinal fluid was normal, and his blood pressure was 200/120.

There can be little doubt that these phenomena, whether gradual or abrupt in their onset, whether temporary or permanent in their results, are directly related to the high arterial tension that is so striking a feature of the clinical background. Yet there are many reasons for believing that the relation is not direct, but mediated by arterial degeneration. In the first place, their liability to occur is by no means directly proportional to the height of the blood pressure. On a recent out-patient day the first two patients seen each of them had systolic pressures of over 300, yet no distal phenomena at all if we except the coronary factor in the total arrhythmia which one of them displayed; while, on the other hand, the third patient was the man of whose claudication I have already spoken, his systolic pressure being round about 200 mm. Hg. Indeed, one may observe all these phenomena—intermittent limp, recurrent paresis, cardiac pain—in persons with normal or even low pressures, provided they have the necessary arterial decay. Perhaps one may ascribe to the hyperpietic syndromes a rather more progressive nature than that of pure atheromatous degeneration. Otherwise there is no obvious difference. It seems clear, therefore, that it is not the high pressure so much as the arterial wear and tear that the pressure has exacted that is responsible for these distal phenomena.

The first proposition, then, that I will ask you to accept is that high arterial tension brings about these distal phenomena in so far as it brings about degeneration of the arterial walls.

We may now turn to discussion of those transient phenomena which, whether they occur in brain, heart or limb, it has been customary to ascribe to arterial spasm.

Let us glance first at some of the clinical evidence. First, then, we note that in the subject of intermittent limp the affected member shows signs of a temporary anæmia. In addition to the lack of arterial pulse, which is usually present between attacks, the skin becomes cold and the limb white. The loss of power and abnormal sensations which go to make up the attack are almost certainly the direct outcome of this failure of the blood to flow into the limb. Secondly, the access of cardiac pain that comes with exertion may be considered. The view that the site of anginal pain lay somewhere in the cardiac wall, and had some relation to its coronary blood supply, has been reinstated by the observations that many of us have been able to contribute as to the association of this kind of pain with coronary infarction. We may therefore go as far as to assert that anginal pain arises in the cardiac wall, and—in all probability—in the muscular tissues. Analysis of a series of case notes showed me that cardiac pain of the ischæmic type, *i.e.* substernal pain coming on during exertion and relieved by its cessation—was complained of in 29 per cent. of the senile cases, 16 per cent. of the hyperpietics, and no less than 43 per cent. of those with cardiac syphilis. For the moment we need go no further than to note that it is precisely in these groups that we encounter aortic and coronary lesions. In syphilis the coronary openings are often so narrow as to be difficult to find, and in the arteriosclerosis of age and of hyperpiesia some degree of change is found either in the suprasigmoid part of the aorta or in the course of the coronaries, or both. All this goes to show that conditions which check the flow of blood into the cardiac muscle furnish the appropriate background for the anginal drama.

In the case of cerebral attacks we can bring to bear less direct evidence to show what happens ; but again analogy, on the one hand with intermittent limp and angina, and on the other with the thrombotic hemiplegias that only differ from the transient attacks in their duration, seems to justify us in a belief that arterial disease causes them by delaying the flow of blood into a localized area of brain. There is also experimental evidence in support of this view, to which I have no time to refer.

All things considered, I believe we are on safe ground in ascribing these temporary failures of function, whether in brain, heart or limb, to a temporary and localized failure of arterial function.

It is customary to think of this arterial failure only in terms of obstruction ; and when actual inspection of arteries, as well as the transitory nature or the phenomena, has proved that in many instances there can have been no organic obstruction, to invoke a hypothetical spasm. It seems to me that we are here still under the dominion of the old mechanical view of the circulation, from which we have been rescued so far as the heart and its work are concerned. That it is a mistake to think of obstruction, whether temporary or permanent, as essential to the provocation of these distal syndromes is suggested by the following scraps of evidence.

In a paper on[5] coronary thrombosis which I read to this Branch a year ago I remarked that in only about one quarter of my cases had there been a long history of attacks of cardiac pain ante-dating that which ushered in and declared the occurrence of infarction in the wall of the heart. The obverse of this is also true. We have all of us seen many patients who have suffered from transient attacks of cardiac pain for years without developing anything more serious. This is hardly what we should expect if the anginal attack were symptomatic of partial, and the thrombotic attack of complete, coronary obstruction. The two would appear more often than they do, I think, as consecutive phases of the same process.

A more direct kind of evidence is afforded by one or two anatomical observations. One of the most striking and suggestive of these was furnished by my surgical colleagues.

Mr. Tasker had asked me to see with him an elderly man, who had for some years been known to suffer from intermittent limp, as well as transient bouts of giddiness. He had a raised tension and tough, deformed arteries. Our immediate concern was how to deal with symptoms of acute intestinal obstruction. I felt that these were possibly due to a mesenteric thrombosis, but thought it wrong to deny him the possibility of surgical relief in case some other cause of obstruction were actually found. Mr. Tasker and Mr. Clifford Moore therefore opened the abdomen and found numerous blackish-blue patches of thickening in the wall of the small bowel, almost exactly like the condition of the herniated bowel in advanced strangulation. The abdominal aorta was enormously dilated in its upper portion, from the diaphragm to just below the renal vessels. His abdomen was closed immediately, and we kept him under the influence of morphia for four or five days. At the end of that time, to our surprise, he had left behind him his abdominal symptoms. Our delight was short-lived, for the next few days saw other arterial catastrophes, ending in a cardiac death which was doubtless due to coronary thrombosis. Yet he gave us the clearest proof that an attack of what one can only call infarction of the bowel, almost certainly arterial in origin, can be recovered from. This must mean return of vitality in the damaged parts. If this had not occurred the obstruction would not have passed off and the peritoneum must have become infected.

Even more convincing, perhaps, was the experience that we had recently at the Bristol General Hospital.

A man aged 59, with chrcnic hyperpiesis of renal origin, died a sudden cardiac death, and was found post-mortem to have a large area of necrosis in the wall of his left ventricle and the septum ventriculorum. In one other case of the kind Dr. Perry's injections of the coronary arteries proved that blood did not enter the necrotic area. In this case, however, we dissected out the arteries entering into the necrotic area, and examined them most carefully, but without discovering any trace of clot or other obstruction to the arterial lumen.

The brain lesions of arterial disease tell a similar story. Being interested in the "hypertensive encephalopathy" alluded to above, not only for its own sake but also because of its possible bearing on this question of the relation of arterial failure to phenomena of organic malnutrition, I asked Dr. Macdonald Critchley, who has been at work on such matters, to give me some information. He referred me to a paper by Foix, Hillemand and Ley,[6] who state that in a series of 56 cases of cerebral softening the arterial obliteration was complete in 12, subtotal in 14, while in 30—or more than half—there remained a lumen of some dimensions.

What can all these observations mean but that quite serious interruptions to the life of a localized area of tissue may be associated with arterial disease, yet without blockage of the artery concerned? At all events, they provoke us to ask whether there is no other arterial function beside that of maintaining an open channel, the failure of which might seriously reduce the blood-flow into the part supplied. Arterial degeneration, whether induced by the over-stress of long-continued hyperpiesis, or more stealthily by the advance of age, is attended by a loss of that elasticity which is one of the chief functions of the arterial tree. For the moment I am not thinking of the muscular elements in the arterial wall so much as of the elastic tissue—a tissue which probably cannot be hypertrophied nor its losses made good by regeneration. The resilience of this element in the walls of the main arteries constitutes the second great propulsive force of the circulation. The arterial tree takes up the blood passed to it by the contracting ventricle, and by its elastic rebound forwards a steady stream of it to the periphery. We are familiar with the belief that loss of this force has, on its proximal side, a deleterious effect on the work of the circulatory pump which, by increasing the cardiac task, calls forth a ventricular hypertrophy. Can it be doubted that on the distal side also there must be effects of arterial failure—loss of efficiency in the local provision of blood, depression of function consequent on that loss, and symptoms indicative of imperfect function ?

Let us test this hypothesis by applying it to the distal syndrome about which we know most—that of the angina of effort, ischæmia cordis, the attack of constricting pain felt behind the sternum during effort and relieved when that effort ceases. It has already been remarked that the three common causes of this syndrome are syphilis of the heart and aorta, cardio-arterial degeneration induced by chronic hyperpiesis, and the cardio-vascular lesions of age. Now, all these three sets of lesions fall with particular severity on that part of the circulatory apparatus which is responsible for the nutrition of the cardiac muscle ; the first part of the thoracic aorta, the aortic semilunar valves, and the coronary arteries. Recent work by Anrep and his associates[7] seems to show that the coronary arteries are filled during ventricular diastole. When the ventricular wall is tightly compressed in systole the coronary arteries share in this compression, so that no blood can enter them. But when the ventricle relaxes in diastole the coronary vessels are open to receive blood from the aorta ; and it is the elastic recoil of the aorta, rendered effective by the closure of the aortic semilunar valves, that fills them. When this elastic recoil is ineffective, even if the coronary vessels be pervious and the aortic valves competent, coronary filling must be inadequate, and particularly when the demands of exertion for an increased coronary flow have to be met, and if the coronary vessels themselves are rigid and inelastic, this inadequacy will become intolerably great. If we view this apparatus—the first part of the aorta with its semilunar valves and its coronary branches —as an organic unit concerned with ensuring a steady supply of blood to the walls of the heart, we shall understand better the frequency with which this apparatus is damaged as a whole by chronic cardiovascular degeneration, and the deadly nature of that damage.

Let me, then, sum up briefly by saying that the distal phenomena of high arterial tension can all of them be ascribed in part to a gradual wearing down of the elasticity of the arterial tree ; in a brief phrase, to progressive arterial failure.

REFERENCES.

[1] Price, *System of Medicine*, 2nd Ed., p. 1,407.

[2] *Bristol Med.-Chir. Jour.*, 1909, xxvii. 15.

[3] *Diseases of the Arteries, including Angina Pectoris*, i. 418 ; London, Macmillan & Co., 1915.

[4] *Arch. Intern. Med.*, 1928, Feb., p. 264.

[5] *Bristol Med.-Chir. Jour.*, 1927, xliv. 249.

[6] *Bull. et MS. des Hôp. d. Paris*, 1927, xliii. 189.

[7] *Heart*, 1927, xiv. 111.

